# Supplementary material for: Hot spot prediction in protein-protein interactions by an ensemble system
Source: BMC Syst Biol. 2018 Dec 31;12(Suppl 9):132. doi: 10.1186/s12918-018-0665-8 (PMC6311905; doi:10.1186/s12918-018-0665-8)
Supplement: Supplementary file 2 — All relevant MATLAB code. A simple Matlab implement of our predictor. (ZIP 19,742 kb) [file 12918_2018_665_MOESM2_ESM.zip › Additional file 2/Read ASA/ASA website/ASEdb/3HFML.htm]

 NetSurfP - Protein Surface Accessibility and Secondary Structure Predictions

  

|  |  |  |
| --- | --- | --- |
|  |  | NetSurfP - Protein Surface Accessibility and Secondary Structure Predictions  Technical University of Denmark |

  


---

```
# For publication of results, please cite:
# A generic method for assignment of reliability scores applied to solvent accessibility predictions.
# Bent Petersen, Thomas Nordahl Petersen, Pernille Andersen, Morten Nielsen and Claus Lundegaard
# BMC Structural Biology 2009, 9:51 doi:10.1186/1472-6807-9-51
#
# Column 1: Class assignment - B for buried or E for Exposed - Threshold: 25% exposure, but not based on RSA
# Column 2: Amino acid
# Column 3: Sequence name
# Column 4: Amino acid number
# Column 5: Relative Surface Accessibility - RSA
# Column 6: Absolute Surface Accessibility
# Column 7: Z-fit score for RSA prediction
# Column 8: Probability for Alpha-Helix
# Column 9: Probability for Beta-strand
# Column 10: Probability for Coil
E D  3HFML                  1    0.797 114.877   0.862   0.003   0.003   0.994
B I  3HFML                  2    0.194  35.927  -1.077   0.003   0.718   0.279
E V  3HFML                  3    0.446  68.566   1.496   0.001   0.900   0.099
B L  3HFML                  4    0.114  20.910   0.678   0.001   0.900   0.099
E T  3HFML                  5    0.448  62.082   0.641   0.002   0.816   0.182
B Q  3HFML                  6    0.135  24.147   0.424   0.004   0.514   0.481
E S  3HFML                  7    0.357  41.852   0.135   0.004   0.138   0.858
E P  3HFML                  8    0.494  70.056  -1.058   0.018   0.019   0.964
E A  3HFML                  9    0.540  59.464  -1.248   0.018   0.088   0.893
E T  3HFML                 10    0.348  48.281  -1.385   0.022   0.552   0.426
B L  3HFML                 11    0.248  45.354  -0.271   0.018   0.846   0.136
E S  3HFML                 12    0.492  57.639  -0.578   0.018   0.846   0.136
B V  3HFML                 13    0.206  31.724   0.111   0.021   0.756   0.223
E T  3HFML                 14    0.423  58.615   0.254   0.004   0.420   0.576
E P  3HFML                 15    0.509  72.213  -0.085   0.004   0.085   0.910
E G  3HFML                 16    0.656  51.604  -1.127   0.005   0.015   0.979
E N  3HFML                 17    0.498  72.834   0.298   0.004   0.138   0.858
E S  3HFML                 18    0.525  61.542   0.938   0.003   0.718   0.279
B V  3HFML                 19    0.062   9.514   0.150   0.001   0.900   0.099
E S  3HFML                 20    0.419  49.072   1.428   0.001   0.959   0.040
B L  3HFML                 21    0.051   9.301   0.357   0.001   0.959   0.040
E S  3HFML                 22    0.324  37.961   0.765   0.001   0.900   0.099
B C  3HFML                 23    0.026   3.707   0.280   0.001   0.900   0.099
E R  3HFML                 24    0.404  92.447   0.965   0.001   0.900   0.099
B A  3HFML                 25    0.165  18.205  -0.835   0.002   0.816   0.182
E S  3HFML                 26    0.368  43.176  -0.902   0.004   0.514   0.481
E Q  3HFML                 27    0.591 105.588  -2.837   0.004   0.085   0.910
E S  3HFML                 28    0.442  51.767  -2.355   0.005   0.045   0.951
E I  3HFML                 29    0.430  79.532  -1.476   0.004   0.085   0.910
E G  3HFML                 30    0.422  33.204  -1.901   0.004   0.085   0.910
B N  3HFML                 31    0.277  40.494  -1.128   0.004   0.138   0.858
B N  3HFML                 32    0.145  21.257   0.231   0.004   0.616   0.381
B L  3HFML                 33    0.024   4.376   0.726   0.001   0.900   0.099
B H  3HFML                 34    0.070  12.697   0.362   0.001   0.900   0.099
B W  3HFML                 35    0.023   5.628   1.114   0.006   0.962   0.032
B Y  3HFML                 36    0.047   9.980   0.507   0.001   0.959   0.040
B Q  3HFML                 37    0.130  23.218   0.957   0.001   0.900   0.099
B Q  3HFML                 38    0.184  32.809   0.544   0.004   0.616   0.381
E K  3HFML                 39    0.368  75.739   1.448   0.004   0.085   0.910
E S  3HFML                 40    0.611  71.644  -0.568   0.005   0.015   0.979
E H  3HFML                 41    0.768 139.681  -2.256   0.003   0.003   0.994
E E  3HFML                 42    0.516  90.163   0.176   0.018   0.019   0.964
E S  3HFML                 43    0.335  39.227  -0.186   0.018   0.047   0.935
B P  3HFML                 44    0.111  15.808  -0.627   0.021   0.279   0.699
E R  3HFML                 45    0.349  79.829   0.723   0.003   0.718   0.279
B L  3HFML                 46    0.112  20.562   0.505   0.001   0.900   0.099
B L  3HFML                 47    0.033   6.024   0.257   0.001   0.959   0.040
B I  3HFML                 48    0.025   4.699   0.206   0.000   0.983   0.017
B K  3HFML                 49    0.099  20.385  -0.176   0.001   0.959   0.040
B Y  3HFML                 50    0.099  21.220  -0.273   0.001   0.900   0.099
B A  3HFML                 51    0.152  16.783  -0.796   0.004   0.514   0.481
B S  3HFML                 52    0.252  29.558  -1.778   0.004   0.197   0.799
E Q  3HFML                 53    0.643 114.858  -1.965   0.005   0.045   0.951
E S  3HFML                 54    0.468  54.838  -1.015   0.005   0.015   0.979
E I  3HFML                 55    0.555 102.657  -2.020   0.018   0.019   0.964
E S  3HFML                 56    0.678  79.438  -1.965   0.018   0.047   0.935
E G  3HFML                 57    0.598  47.102  -1.195   0.018   0.047   0.935
B I  3HFML                 58    0.229  42.328   0.053   0.018   0.088   0.893
E P  3HFML                 59    0.526  74.668  -0.697   0.004   0.085   0.910
E S  3HFML                 60    0.423  49.599  -2.061   0.004   0.197   0.799
B R  3HFML                 61    0.288  66.021  -0.508   0.004   0.514   0.481
B F  3HFML                 62    0.096  19.327  -1.451   0.003   0.718   0.279
E S  3HFML                 63    0.406  47.525  -0.847   0.003   0.718   0.279
E G  3HFML                 64    0.348  27.411  -1.920   0.004   0.616   0.381
E S  3HFML                 65    0.429  50.337  -1.359   0.004   0.514   0.481
E G  3HFML                 66    0.544  42.836  -1.546   0.005   0.262   0.733
E S  3HFML                 67    0.521  61.026  -1.489   0.004   0.138   0.858
E G  3HFML                 68    0.550  43.246  -1.308   0.004   0.138   0.858
E T  3HFML                 69    0.435  60.334  -0.989   0.005   0.336   0.660
E D  3HFML                 70    0.394  56.790  -0.112   0.003   0.718   0.279
B F  3HFML                 71    0.084  16.859  -0.216   0.001   0.900   0.099
E T  3HFML                 72    0.264  36.672   0.036   0.001   0.900   0.099
B L  3HFML                 73    0.048   8.770   0.236   0.001   0.900   0.099
E S  3HFML                 74    0.306  35.840   0.494   0.001   0.900   0.099
B I  3HFML                 75    0.068  12.598  -0.182   0.002   0.816   0.182
E N  3HFML                 76    0.403  58.999   0.279   0.004   0.616   0.381
E S  3HFML                 77    0.455  53.303  -1.109   0.005   0.336   0.660
B V  3HFML                 78    0.281  43.220  -0.619   0.019   0.141   0.840
E E  3HFML                 79    0.479  83.646   0.291   0.020   0.205   0.775
E T  3HFML                 80    0.411  56.950  -0.751   0.056   0.142   0.802
E E  3HFML                 81    0.507  88.625  -1.065   0.056   0.142   0.802
E D  3HFML                 82    0.498  71.819  -0.966   0.020   0.205   0.775
E F  3HFML                 83    0.359  72.112  -0.687   0.022   0.359   0.619
B G  3HFML                 84    0.266  20.966  -1.020   0.004   0.514   0.481
E M  3HFML                 85    0.324  64.752  -0.022   0.004   0.616   0.381
B Y  3HFML                 86    0.076  16.327   0.550   0.002   0.816   0.182
B F  3HFML                 87    0.162  32.554   0.848   0.002   0.816   0.182
B C  3HFML                 88    0.037   5.237  -0.754   0.002   0.816   0.182
B Q  3HFML                 89    0.154  27.540  -0.236   0.018   0.846   0.136
B Q  3HFML                 90    0.105  18.717  -1.219   0.023   0.655   0.322
B S  3HFML                 91    0.278  32.628  -0.833   0.021   0.451   0.528
E N  3HFML                 92    0.345  50.567  -1.274   0.020   0.205   0.775
E S  3HFML                 93    0.476  55.846  -2.248   0.056   0.142   0.802
E W  3HFML                 94    0.364  87.422  -1.097   0.056   0.142   0.802
E P  3HFML                 95    0.338  47.891  -1.896   0.052   0.084   0.864
B Y  3HFML                 96    0.292  62.379  -1.062   0.052   0.084   0.864
E T  3HFML                 97    0.414  57.450  -1.750   0.052   0.084   0.864
E F  3HFML                 98    0.407  81.785  -1.290   0.052   0.084   0.864
E G  3HFML                 99    0.334  26.317  -1.831   0.056   0.142   0.802
E G  3HFML                100    0.335  26.396  -1.032   0.064   0.216   0.721
E G  3HFML                101    0.368  28.922  -1.102   0.064   0.216   0.721
E T  3HFML                102    0.364  50.542  -1.058   0.021   0.279   0.699
E K  3HFML                103    0.328  67.408  -0.871   0.069   0.386   0.545
E L  3HFML                104    0.316  57.896  -0.842   0.069   0.386   0.545
E E  3HFML                105    0.349  60.935  -1.092   0.066   0.296   0.638
E I  3HFML                106    0.328  60.754  -1.322   0.066   0.296   0.638
E K  3HFML                107    0.412  84.666  -1.029   0.066   0.296   0.638
E R  3HFML                108    0.464 106.233  -1.460   0.066   0.296   0.638
E A  3HFML                109    0.467  51.441  -1.337   0.064   0.216   0.721
E D  3HFML                110    0.462  66.589  -1.184   0.064   0.216   0.721
E A  3HFML                111    0.369  40.609  -1.028   0.056   0.142   0.802
E A  3HFML                112    0.407  44.807  -1.466   0.056   0.142   0.802
E P  3HFML                113    0.417  59.144  -1.145   0.056   0.142   0.802
E T  3HFML                114    0.447  62.013  -1.626   0.118   0.150   0.732
E V  3HFML                115    0.360  55.347  -1.049   0.125   0.227   0.648
E S  3HFML                116    0.487  57.123  -1.716   0.125   0.227   0.648
E I  3HFML                117    0.440  81.437  -1.697   0.118   0.150   0.732
E F  3HFML                118    0.378  75.905  -1.607   0.056   0.142   0.802
E P  3HFML                119    0.326  46.217  -1.185   0.113   0.087   0.800
E P  3HFML                120    0.433  61.386  -1.381   0.118   0.150   0.732
E S  3HFML                121    0.382  44.829  -0.820   0.125   0.227   0.648
E S  3HFML                122    0.327  38.313  -1.703   0.216   0.235   0.548
E E  3HFML                123    0.341  59.643  -1.045   0.216   0.235   0.548
E Q  3HFML                124    0.361  64.492  -2.093   0.216   0.235   0.548
E L  3HFML                125    0.328  60.002  -1.411   0.199   0.152   0.649
E T  3HFML                126    0.494  68.448  -1.291   0.113   0.087   0.800
E S  3HFML                127    0.536  62.831  -1.258   0.053   0.043   0.903
E G  3HFML                128    0.521  41.034  -1.527   0.018   0.088   0.893
E G  3HFML                129    0.400  31.496  -0.864   0.005   0.262   0.733
B A  3HFML                130    0.113  12.431  -1.127   0.003   0.718   0.279
B S  3HFML                131    0.200  23.452   0.457   0.001   0.900   0.099
B V  3HFML                132    0.040   6.087  -0.112   0.001   0.900   0.099
B V  3HFML                133    0.075  11.512   0.202   0.001   0.959   0.040
B C  3HFML                134    0.016   2.204   1.220   0.001   0.900   0.099
B F  3HFML                135    0.094  18.806  -1.045   0.001   0.900   0.099
B L  3HFML                136    0.031   5.731  -1.081   0.002   0.816   0.182
B N  3HFML                137    0.144  21.155  -0.574   0.003   0.718   0.279
B N  3HFML                138    0.207  30.290  -1.346   0.004   0.420   0.576
E F  3HFML                139    0.363  72.874  -2.137   0.004   0.197   0.799
B Y  3HFML                140    0.303  64.815  -1.597   0.004   0.085   0.910
E P  3HFML                141    0.415  58.903  -2.480   0.004   0.085   0.910
E K  3HFML                142    0.514 105.709  -2.418   0.004   0.138   0.858
E D  3HFML                143    0.411  59.283  -1.175   0.004   0.197   0.799
B I  3HFML                144    0.071  13.117  -1.114   0.005   0.336   0.660
B N  3HFML                145    0.211  30.846  -0.368   0.003   0.718   0.279
B V  3HFML                146    0.053   8.192  -0.220   0.002   0.816   0.182
B K  3HFML                147    0.155  31.986  -0.183   0.011   0.918   0.071
B W  3HFML                148    0.078  18.783  -0.831   0.018   0.846   0.136
B K  3HFML                149    0.255  52.515   0.058   0.021   0.756   0.223
E I  3HFML                150    0.356  65.804   0.617   0.004   0.420   0.576
E D  3HFML                151    0.679  97.887  -0.468   0.005   0.015   0.979
E G  3HFML                152    0.741  58.332  -2.031   0.005   0.015   0.979
E S  3HFML                153    0.643  75.371  -0.516   0.020   0.205   0.775
E E  3HFML                154    0.512  89.411  -0.938   0.020   0.205   0.775
E R  3HFML                155    0.291  66.662  -0.609   0.019   0.141   0.840
E Q  3HFML                156    0.545  97.408  -1.159   0.020   0.205   0.775
E N  3HFML                157    0.370  54.153  -1.390   0.022   0.359   0.619
B G  3HFML                158    0.281  22.107  -1.602   0.004   0.514   0.481
B V  3HFML                159    0.245  37.610  -0.934   0.002   0.816   0.182
B L  3HFML                160    0.223  40.905  -0.543   0.011   0.918   0.071
E N  3HFML                161    0.406  59.497  -0.787   0.018   0.846   0.136
B S  3HFML                162    0.211  24.753  -1.501   0.022   0.552   0.426
E W  3HFML                163    0.345  82.924  -0.516   0.021   0.451   0.528
E T  3HFML                164    0.488  67.658  -0.981   0.021   0.279   0.699
E D  3HFML                165    0.388  55.968  -1.102   0.018   0.088   0.893
E Q  3HFML                166    0.569 101.570  -1.971   0.018   0.088   0.893
E D  3HFML                167    0.590  85.091  -1.851   0.018   0.088   0.893
E S  3HFML                168    0.591  69.242  -1.517   0.018   0.047   0.935
E K  3HFML                169    0.508 104.434  -0.646   0.018   0.019   0.964
E D  3HFML                170    0.659  95.005  -1.879   0.005   0.015   0.979
E S  3HFML                171    0.469  55.025  -1.811   0.005   0.045   0.951
E T  3HFML                172    0.340  47.186   0.152   0.004   0.420   0.576
B Y  3HFML                173    0.077  16.412  -1.379   0.003   0.718   0.279
B S  3HFML                174    0.105  12.341  -1.126   0.002   0.816   0.182
B M  3HFML                175    0.061  12.146  -1.171   0.001   0.900   0.099
B S  3HFML                176    0.126  14.779  -0.749   0.001   0.959   0.040
B S  3HFML                177    0.040   4.641  -1.587   0.001   0.959   0.040
B T  3HFML                178    0.184  25.493  -0.168   0.001   0.959   0.040
B L  3HFML                179    0.040   7.379  -0.180   0.001   0.900   0.099
E T  3HFML                180    0.291  40.376   0.643   0.001   0.900   0.099
B L  3HFML                181    0.067  12.231   0.197   0.002   0.816   0.182
E T  3HFML                182    0.364  50.515   0.827   0.005   0.336   0.660
E K  3HFML                183    0.460  94.560  -0.838   0.020   0.205   0.775
E D  3HFML                184    0.541  77.929  -0.978   0.020   0.205   0.775
E E  3HFML                185    0.533  93.168   0.710   0.020   0.205   0.775
B Y  3HFML                186    0.199  42.569  -0.819   0.018   0.088   0.893
E E  3HFML                187    0.671 117.154   0.492   0.005   0.045   0.951
E R  3HFML                188    0.642 147.132   0.758   0.005   0.015   0.979
E H  3HFML                189    0.602 109.449  -0.412   0.005   0.045   0.951
E N  3HFML                190    0.441  64.518  -0.000   0.005   0.336   0.660
E S  3HFML                191    0.442  51.802   1.555   0.002   0.816   0.182
B Y  3HFML                192    0.064  13.613   1.064   0.001   0.959   0.040
B T  3HFML                193    0.270  37.435   1.766   0.001   0.959   0.040
B C  3HFML                194    0.021   2.962   0.957   0.001   0.959   0.040
B E  3HFML                195    0.212  37.054   1.242   0.001   0.959   0.040
B A  3HFML                196    0.069   7.637  -0.042   0.001   0.959   0.040
E T  3HFML                197    0.357  49.460   0.543   0.001   0.959   0.040
B H  3HFML                198    0.134  24.356   0.342   0.002   0.816   0.182
E K  3HFML                199    0.602 123.831  -0.144   0.004   0.197   0.799
E T  3HFML                200    0.645  89.461  -1.801   0.005   0.045   0.951
E S  3HFML                201    0.443  51.931  -0.824   0.005   0.045   0.951
E T  3HFML                202    0.618  85.730  -0.108   0.005   0.045   0.951
E S  3HFML                203    0.556  65.116   0.658   0.019   0.141   0.840
E P  3HFML                204    0.391  55.554   0.415   0.022   0.359   0.619
B I  3HFML                205    0.130  24.124  -0.444   0.003   0.718   0.279
E V  3HFML                206    0.535  82.168   0.452   0.001   0.900   0.099
E K  3HFML                207    0.291  59.859   1.391   0.001   0.900   0.099
E S  3HFML                208    0.469  54.943   0.110   0.001   0.900   0.099
B F  3HFML                209    0.090  18.083   0.851   0.002   0.816   0.182
E N  3HFML                210    0.495  72.512   1.737   0.004   0.514   0.481
E R  3HFML                211    0.456 104.470   0.707   0.019   0.141   0.840
E N  3HFML                212    0.661  96.800  -0.960   0.018   0.088   0.893
E E  3HFML                213    0.637 111.301   1.628   0.018   0.088   0.893
E C  3HFML                214    0.436  61.214  -1.238   0.003   0.003   0.994

Explain the output.  Go back.
```

---
